# Neuropathology in the North American sudden unexpected death in epilepsy registry

**DOI:** 10.1093/braincomms/fcab192

**Published:** 2021-08-23

**Authors:** Dominique F Leitner, Arline Faustin, Chloe Verducci, Daniel Friedman, Christopher William, Sasha Devore, Thomas Wisniewski, Orrin Devinsky

**Affiliations:** 1 Comprehensive Epilepsy Center, NYU Grossman School of Medicine, New York, NY, USA; 2 Department of Neurology, NYU Langone Health and School of Medicine, New York, NY, USA; 3 Center for Cognitive Neurology, NYU Langone Health and School of Medicine, New York, NY, USA; 4 Department of Pathology, NYU Langone Health and School of Medicine, New York, NY, USA; 5 Department of Psychiatry, NYU Langone Health and School of Medicine, New York, NY, USA

**Keywords:** SUDEP, neuropathology, epilepsy, seizures

## Abstract

Sudden unexpected death in epilepsy is the leading category of epilepsy-related death and the underlying mechanisms are incompletely understood. Risk factors can include a recent history and high frequency of generalized tonic-clonic seizures, which can depress brain activity postictally, impairing respiration, arousal and protective reflexes. Neuropathological findings in sudden unexpected death in epilepsy cases parallel those in other epilepsy patients, with no implication of novel structures or mechanisms in seizure-related deaths. Few large studies have comprehensively reviewed whole brain examination of such patients. We evaluated 92 North American Sudden unexpected death in epilepsy Registry cases with whole brain neuropathological examination by board-certified neuropathologists blinded to the adjudicated cause of death, with an average of 16 brain regions examined per case. The 92 cases included 61 sudden unexpected death in epilepsy (40 definite, 9 definite plus, 6 probable, 6 possible) and 31 people with epilepsy controls who died from other causes. The mean age at death was 34.4 years and 65.2% (60/92) were male. The average age of death was younger for sudden unexpected death in epilepsy cases than for epilepsy controls (30.0 versus 39.6 years; *P* = 0.006), and there was no difference in sex distribution respectively (67.3% male versus 64.5%, *P* = 0.8). Among sudden unexpected death in epilepsy cases, earlier age of epilepsy onset positively correlated with a younger age at death (*P* = 0.0005) and negatively correlated with epilepsy duration (*P* = 0.001). Neuropathological findings were identified in 83.7% of the cases in our cohort. The most common findings were dentate gyrus dysgenesis (sudden unexpected death in epilepsy 50.9%, epilepsy controls 54.8%) and focal cortical dysplasia (FCD) (sudden unexpected death in epilepsy 41.8%, epilepsy controls 29.0%). The neuropathological findings in sudden unexpected death in epilepsy paralleled those in epilepsy controls, including the frequency of total neuropathological findings as well as the specific findings in the dentate gyrus, findings pertaining to neurodevelopment (e.g. FCD, heterotopias) and findings in the brainstem (e.g. medullary arcuate or olivary dysgenesis). Thus, like prior studies, we found no neuropathological findings that were more common in sudden unexpected death in epilepsy cases. Future neuropathological studies evaluating larger sudden unexpected death in epilepsy and control cohorts would benefit from inclusion of different epilepsy syndromes with detailed phenotypic information, consensus among pathologists particularly for more subjective findings where observations can be inconsistent, and molecular approaches to identify markers of sudden unexpected death in epilepsy risk or pathogenesis.

## Teaser

Leitner et al. report no neuropathological findings more common in sudden unexpected death in epilepsy (SUDEP) compared to non-SUDEP epilepsy cases in the North American SUDEP Registry (NASR) cohort. Future studies may identify findings associated with SUDEP risk from larger SUDEP and control cohorts with detailed clinical information.

## Graphical Abstract

**Graphical Abstract:**
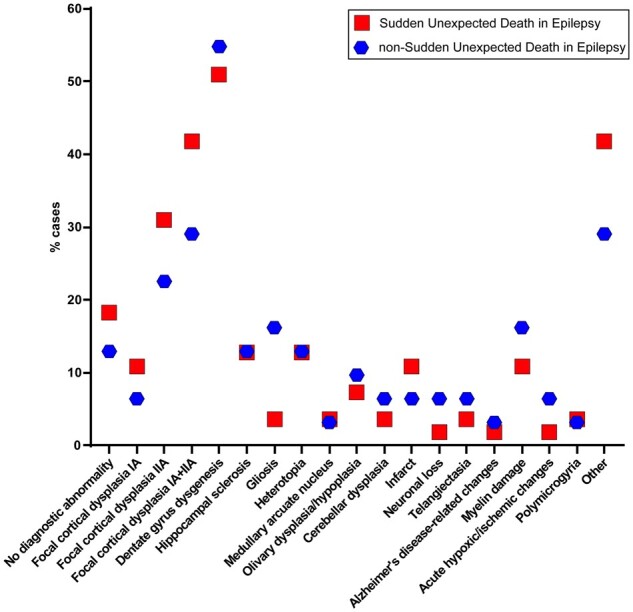

## Introduction

Sudden unexpected death in epilepsy (SUDEP) is the leading category of epilepsy-related death, affecting ∼1/1000 epilepsy patients each year.^1^ Most SUDEP cases are unwitnessed, occur during sleep, and subjects are found prone. Although epidemiological, risk factor, mechanistic, and preventative studies have advanced our understanding of SUDEP, the underlying mechanisms are not well understood.^2–4^ SUDEP risk factors include recent occurrence and high frequency of generalized tonic-clonic seizures (GTCS), living alone or unsupervised at night, polytherapy, epilepsy duration, young disease onset, sex, lamotrigine therapy and anti-seizure medication (ASM) non-adherence.^5–9^ However, many questions remain unanswered, including the role of risk factor combinations, why SUDEP is more common in sleep, and what types and intensities of postictal stimulation can prevent some SUDEP cases. SUDEP usually occurs shortly after a GTCS,^6^ but there is little understanding why a person’s 20th or 200th GTCS is deadly while the earlier ones were not, or why some die during their second or third GTCS while others survive many hundreds.

Animal models and human studies support that abnormalities in respiration, arousal and parasympathetic hyperactivity contribute to SUDEP pathogenesis based on EEG, ECG, respiratory and imaging studies.^1^^,^^10–14^ After a GTCS, a prolonged postictal EEG suppression (PGES) may occur, in which many patients are unresponsive, have impaired respiration and autonomic function (e.g. postictal central apnea^15–17^ and hypoxia^18^^,^^19^), and diffusely attenuated EEG that reflects profound cerebral dysfunction.^20^^,^^21^ Suppression of respiration and arousal during PGES, combined with a prone, face-down position increased SUDEP risk in some,^20^ but not in all studies.^22^ Furthermore, structural changes in brain autonomic and arousal networks due to chronic epilepsy may potentiate impaired postictal arousal, respiration and cardio-regulatory dysfunction that increase SUDEP risk. Epilepsy-related, long-term, progressive neural injury on structural and functional imaging includes atrophy, microstructural damage and hypometabolism in and beyond the epileptogenic zone.^23–29^ These progressive changes affect subcortical and cortical volumes,^14^^,^^23^^,^^25^^,^^30^ as well as the hippocampus.^31^^,^^32^

Neuropathological, immunohistochemical and molecular studies have identified few differences between SUDEP victims and epilepsy patients who died from other causes,^33–39^ with the particular exception of one study describing medullary changes in glial populations among SUDEP cases.^40^ A review of 145 SUDEP cases found similar neuropathological findings when compared to three smaller forensic series,^4^ and indicated no specific finding that distinguish SUDEP versus people with epilepsy (PWE) cases.^33^ However, few large studies have comprehensively reviewed extensive neuropathological examination by board-certified neuropathologists.

We performed whole brain neuropathological examination of epilepsy cases in the North American SUDEP Registry (NASR) and evaluated whether clinical variables were associated with SUDEP status or neuropathological findings. We compared the frequency of neuropathological findings in SUDEP and PWE overall, including review of findings in the dentate gyrus, findings pertaining to neurodevelopment and findings in brainstem autonomic centres.

## Materials and methods

### Case selection

Cases include epilepsy patients who died from SUDEP or other causes enrolled in NASR with approval by the New York University Grossman School of Medicine Institutional Review Board (IRB). NASR enrollment began in October 2011,^2^ with all next-of-kin providing written informed consent. Cases were reviewed through February 2020. Causes of death were classified into non-SUDEP epilepsy and SUDEP (definite SUDEP, definite SUDEP plus, probable SUDEP, possible SUDEP, near SUDEP and presumed SUDEP).^2^^,^^41^ Cases were selected with inclusion criteria of whole brain neuropathology exam and included all non-SUDEP and SUDEP cases. We excluded ‘presumed SUDEP’ cases with insufficient information to determine SUDEP group and ‘near SUDEP’.^2^ PWE included the following causes of death: blunt force injury, acute intoxication/toxicity, aspiration, pneumonia, drowning, sepsis, ruptured aortic dissection and suicide. The demographic and clinical information of our 92 cases are summarized in Tables 1 and 2, Supplementary Table S1.

**Table 1:** Case history

Study group	*n*	Sex M/F	Mean age at death (years)	Mean age of onset (years)	Mean disease duration (years)	Mean brain weight (g)
PWE	31	20/11	39.6 ± 17.6	15.2 ± 13.7	21.0 ± 17.9	1363 ± 241
Possible SUDEP	6	3/3	48.2 ± 11.5	30.8 ± 22.7	21.6 ± 19.1	1442 ± 98
Probable SUDEP	6	3/3	33.8 ± 17.5	17.1 ± 19.3	22.6 ± 22.4	1318 ± 265
Definite SUDEP	40	29/11	28.0 ± 12.0	13.7 ± 10.4	14.4 ± 11.6	1408 ± 157
			Adj. *P* = 0.01 (PWE)			
			Adj. *P* = 0.02 (possible-SUDEP)			
Definite SUDEP plus	9	5/4	36.4 ± 16.1	11.9 ± 16.0	20.9 ± 17.3	1382 ± 126
Total SUDEP^a^	55	37/18	30.0 ± 13.5	13.7 ± 11.9	16.1 ± 13.6	1394 ± 166
			*P* = 0.006 (PWE)			

Known information is included: mean age of onset (*n* = 71), mean disease duration (*n* = 69), mean brain weight (*n* = 91). One-way ANOVA with Tukey’s *post hoc* test performed among all groups except total SUDEP. Total SUDEP overall versus PWE by two-tailed *t*-test.

adj. *P* = adjusted *P*-value; *n* = number of cases in each group.

a Excludes possible SUDEP.

**Table 2:** Circumstances of death

	% known cases				Known cases (*n*)	Unknown cases (*n*)
Position						
	Prone	Supine	Side	Other		
PWE	15.8	**47.4**	15.8	21.1	19	12
Possible SUDEP	0.0	**83.3**	0.0	16.7	6	0
Probable SUDEP	**66.7**	33.3	0.0	0.0	3	3
Definite SUDEP	**63.2**	21.1	0.0	15.8	38	2
Definite SUDEP plus	14.3	**42.9**	14.3	28.6	7	2
Total SUDEP^a^	**56.3**	25.0	2.1	16.7	48	7
Found in bed						
	Yes	No				
PWE	36.4	**63.6**			22	9
Possible SUDEP	**60.0**	40.0			5	1
Probable SUDEP	**60.0**	40.0			5	1
Definite SUDEP	**69.7**	30.3			33	7
Definite SUDEP plus	42.9	**57.1**			7	2
Total SUDEP^a^	**64.4**	35.6			45	10
Awake/asleep						
	Asleep	Awake				
PWE	40.0	**60.0**			20	11
Possible SUDEP	**66.7**	33.3			6	0
Probable SUDEP	**60.0**	40.0			5	1
Definite SUDEP	**71.4**	28.6			35	5
Definite SUDEP plus	42.9	**57.1**			7	2
Total SUDEP^a^	**66.0**	34.0			47	8
Death witnessed						
	Yes	No				
PWE	17.9	**82.1**			28	3
Possible SUDEP	16.7	**83.3**			6	0
Probable SUDEP	33.3	**66.7**			6	0
Definite SUDEP	2.5	**97.5**			40	0
Definite SUDEP plus	0.0	**100.0**			8	1
Total SUDEP^a^	5.6	**94.4**			54	1
Seizure witnessed						
	Yes	No				
PWE	10.7	**89.3**			28	3
Possible SUDEP	0.0	**100.0**			5	1
Probable SUDEP	16.7	**83.3**			6	0
Definite SUDEP	10.0	**90.0**			40	0
Definite SUDEP plus	25.0	**75.0**			8	1
Total SUDEP^a^	13.0	**87.0**			54	1
ASM adherence						
	Yes	No				
PWE	44.4	**55.6**			9	22
Possible SUDEP	0.0	**100.0**			3	3
Probable SUDEP	**75.0**	25.0			4	2
Definite SUDEP	47.4	**52.6**			25 (19 on ASMs)	15
Definite SUDEP plus	**66.7**	33.3			7 (6 on ASMs)	2
Total SUDEP^a^	**55.2**	44.8			36	19

Bolded are the most frequent circumstances for the percentage of cases with known information in each group.

All known cases on ASMs unless otherwise indicated.

ASM, anti-seizure medication; *n* = number of cases.

a Excludes possible SUDEP.

### Clinical history

NASR routinely acquired all available medical records, including MRI, EEG, video EEG and ECG reports and original data when available. NASR neurologists and cardiologists adjudicated seizure types, epilepsy syndrome and diagnostic test findings (EEG/video EEG, MRI, ECG) to confirm diagnosis.^2^ Seizure types were categorized as focal, generalized or mixed. Lifetime GTCS history was obtained from family interviews and medical records.^2^ Specific epilepsy syndromes (e.g. juvenile myoclonic, Dravet) were not analysed, as only 20 cases had this information. Circumstances of death are summarized in Table 2, including position, whether the decedent was found in bed, was awake or asleep, death was witnessed, seizure was witnessed in the preceding period immediately before death, and whether ASM adherence shortly before death was known. The number of ASMs prescribed at the time of death (*n* = 78 cases) are summarized in Supplementary Table S1. Autopsy reports were reviewed for pulmonary and cardiac findings (*n* = 79 cases).

### Neuropathology

All neuropathology examinations were performed at NYU by three board-certified neuropathologists (T.W., A.F. and C.W.) that were blinded to the adjudicated cause of death (COD). Whole brains were fixed ≥2 weeks before arrival at NYU for cases originating outside NYU. Fixed brain weight was available for 91/92 cases (1 possible SUDEP case included partial brain weight and was excluded in weight analyses). Macroscopic and microscopic examination was performed for all 92 cases according to current international consensus criteria.^42–45^ After gross neuropathological review, each brain was sampled to generate 13–20 formalin fixed paraffin embedded (FFPE) blocks (mean, 16 per case) and sections were stained with luxol fast blue, haematoxylin and eosin (LFB/H&E). Additional immunostaining was performed to evaluate dysmorphic neurons in focal cortical dysplasia (FCD) type IIA, using standard methods targeting Ser240/244 phosphorylated ribosomal protein S6 (phospho-S6; 1:250, Cell Signaling #5364) followed by avidin-biotin peroxidase and DAB chromogen counterstained with haematoxylin as described previously.^46^ Variation in the number of FFPE blocks reflected available brain regions of a previously cut brain, additional sampling for lesions of interest, and increased sampling done in later cases to include hippocampi and additional medullary sections (*n* = 40).

### Whole slide scanning

Representative neuropathological findings on the LFB/H&E and phospho-S6 stained sections from neuropathology review were depicted after whole slide scanning. Images were acquired at 20× magnification on NanoZoomer HT2 (Hamamatsu) and Leica Aperio Versa 8 microscopes.

### Statistical analyses

Statistical analyses were performed with GraphPad Prism (version 8) and using the R environment (http://www.r-project.org/ Last accessed February 2021). To evaluate clinical data (i.e. age at death, brain weight, number of neuropathological findings), a one-way ANOVA with a Tukey’s *post hoc* test was performed among groups and a two-tailed *t* test was used in pairwise comparisons (i.e. PWE and SUDEP). To determine the frequency of neuropathologic findings, Fisher’s exact tests were performed. Correlation analyses of clinical data were calculated with a Pearson correlation. In each statistical analysis, *P*-value <0.05 was considered significant.

### Data availability

The raw data for this study are available to qualified researchers from the corresponding author upon request.

## Results

### Case history

Among the 92 NASR cases, average age at death was 34.4 years (range 2–66 years; median 32.5; SUDEP mean, 30.0 years; PWE mean, 39.6 years); and 65.2% (60/92) of cases were male (SUDEP 67.3% male; PWE 64.5% male). Table 1 summarizes cases by SUDEP subgroup and related case history. Definite SUDEP cases were younger at death than PWE (*P* = 0.01) and possible SUDEP (*P* = 0.02); Table 1, Supplementary Fig. S1A. SUDEP cases were younger than PWE (*P* = 0.006). There was no significant difference between mean age of epilepsy onset or disease duration among the SUDEP subgroups (one-way ANOVA, Table 1). Among all epilepsy cases, there was a positive correlation between age at death and (i) age of epilepsy onset (*P* < 0.0001, *R*^2^ = 0.2, Pearson correlation) and (ii) disease duration (*P* = 0.0003, *R*^2^ = 0.2, Supplementary Fig. S1B and C). There was also a negative correlation of age of epilepsy onset and epilepsy duration in epilepsy overall (*P* < 0.0001, *R*^2^ = 0.2, Supplementary Fig. S1D). For SUDEP cases, there was a positive correlation between age at death and age of epilepsy onset for total SUDEP (*P* = 0.0005, *R*^2^ = 0.2) but not PWE (Supplementary Fig. S1B). With an earlier age at death in SUDEP, total SUDEP cases tend to die at a 2.2-fold faster rate after epilepsy onset overall than PWE but this is not significant (*P* = 0.3). Age at death and disease duration were positively correlated in PWE (*P* = 0.04, *R*^2^ = 0.2) and SUDEP (*P* = 0.007, *R*^2^ = 0.2); and were similar between PWE and SUDEP (*P* > 0.9, Supplementary Fig. S1C). Furthermore, age of epilepsy onset correlated with disease duration in both PWE (*P* = 0.009, *R*^2^ = 0.4) and SUDEP (*P* = 0.001, *R*^2^ = 0.2); and were similar between PWE and SUDEP (*P* = 0.4, Supplementary Fig. S1D).

### Circumstances of death

Circumstances of death are summarized in Table 2. Among SUDEP cases, 56.3% were found prone, 64.4% were in bed, 66.0% were presumed asleep, 94.4% were unwitnessed deaths, seizure was not witnessed before death (87.0%) and were ASM adherent (55.2%). Among the PWE cases, 47.4% were found supine, 63.6% were not in bed, 60.0% were awake, death was unwitnessed in 82.1%, no seizure was witnessed before death in 89.3% and 55.6% were not ASM adherent. Among the 78 PWE and SUDEP cases with ASM data, there were similar levels of ASM adherence (*P* = 0.7). There was no significant difference between PWE and total SUDEP for cases with ≥2 or ≥3 ASMs (Supplementary Table S1).

### Neuropathological findings in PWE and SUDEP

Brain weight was similar in PWE and SUDEP groups (Table 1). Among all epilepsy cases, there was a negative correlation of brain weight and age at death (*P* = 0.03, *R*^2^ = 0.05), but not with age of onset (*P* = 0.7, *R*^2^ = 0.002) nor with disease duration (*P* = 0.08, *R*^2^ = 0.05, Supplementary Fig. S1E–G). When evaluating in PWE and all SUDEP cases, there was a negative correlation of brain weight and disease duration in SUDEP (*P* < 0.05, *R*^2^ = 0.09; Supplementary Fig. S1G), but no correlation of disease duration in PWE. There was no correlation of brain weight with age at death or age of onset in PWE or SUDEP.

**Figure 1:**
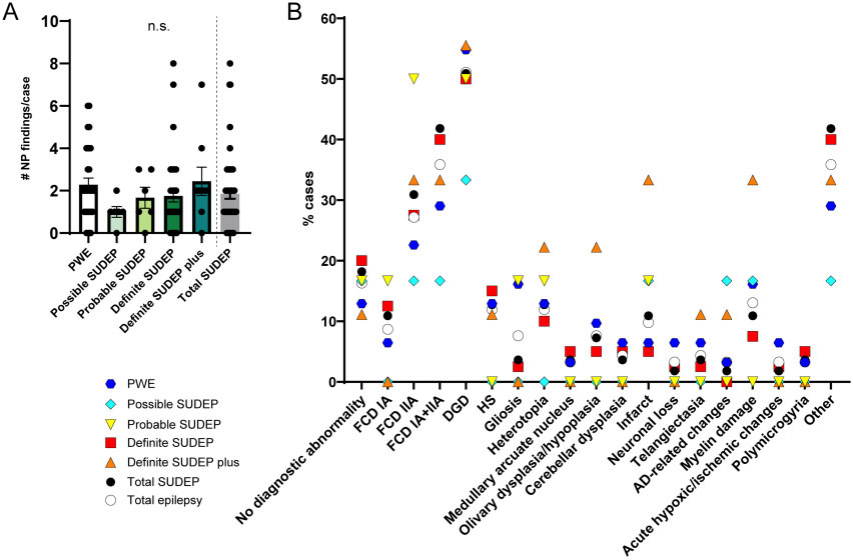
**Neuropathology findings in PWE and SUDEP subgroups**. (**A**) The number of neuropathological findings per case were not significantly different in the total SUDEP group when compared to PWE (two-tailed *t*-test), nor among the SUDEP subgroups (one-way ANOVA Tukey’s *post hoc* test). The mean is depicted with errors bars representing SEM. (**B**) The percent of cases with neuropathology findings in each group are depicted for the diagnoses described on the *x*-axis. The most frequent finding overall in epilepsy was dentate gyrus dysgenesis (DGD) at 51.1% and focal cortical dysplasia (FCD) at 35.9% of cases. For the total SUDEP group, the most frequent findings were DGD in 50.9% of cases and FCD in 41.8% of cases. For the PWE group, the most frequent findings were DGD in 54.8% of cases and FCD in 29.0% of cases. For all SUDEP subgroups, the most frequent finding was DGD. When comparing the total SUDEP and PWE groups, there was no significant difference in the frequency of each neuropathological finding (Fisher’s exact test).

At least one neuropathological finding was observed in 83.7% (77/92) of all cases: 87.1% (27/31) PWE and 81.8% (45/55) SUDEP cases (Fig. 1A). The number of findings per case was similar for PWE and SUDEP groups (Fig. 1A). Among all epilepsy (PWE/SUDEP) cases (Fig. 1B), dentate gyrus dysgenesis [DGD; 54.8% (17/31) PWE, 50.9% (28/55) SUDEP] was most common followed by FCD [29.0% (9/31) PWE, 41.8% (23/55) SUDEP]. There were no significant differences in neuropathological findings comparing PWE and SUDEP (Table 3). For SUDEP subgroups, FCD was most common: 66.7% (4/6) of probable SUDEP and 40.0% (16/40) of definite SUDEP cases. For the possible SUDEP and definite SUDEP plus subgroups, multiple findings were distributed across these small subgroups. The study lacked power to examine statistical differences across SUDEP subgroups.

**Table 3:** Neuropathology in PWE and total SUDEP cases

Neuropathology finding	PWE (%)	PWE (*n*)	Total SUDEP (%)	Total SUDEP (*n*)	*P*-value
No diagnostic abnormality	12.9	4	18.2	10	0.7
FCD IA + IIA	29.0	9	41.8	23	0.3
FCD IA	6.5	2	10.9	6	0.7
FCD IIA	22.6	7	30.9	17	0.5
DGD	54.8	17	50.9	28	0.8
HS	12.9	4	12.7	7	>0.9
Gliosis	16.1	5	3.6	2	0.09
Heterotopia	12.9	4	12.7	7	>0.9
Medullary arcuate nucleus dysgenesis	3.2	1	3.6	2	>0.9
Medullary olivary dysplasia/hypoplasia	9.7	3	7.3	4	0.7
Cerebellar dysplasia	6.5	2	3.6	2	0.6
Infarct	6.5	2	10.9	6	0.7
Neuronal loss	6.5	2	1.8	1	0.3
Telangiectasia	6.5	2	3.6	2	0.6
AD-related changes	3.2	1	1.8	1	>0.9
Myelin damage	16.1	5	10.9	6	0.5
Acute hypoxic/ischaemic changes	6.5	2	1.8	1	0.3
Polymicrogyria	3.2	1	3.6	2	>0.9
Other	25.8	8	14.5	8	0.3

PWE (*n* = 31); total SUDEP (*n* = 55) excludes possible SUDEP cases.

Fisher’s exact test performed for each neuropathological finding.

%, percent of cases; *n* = number of cases.

**Figure 2:**
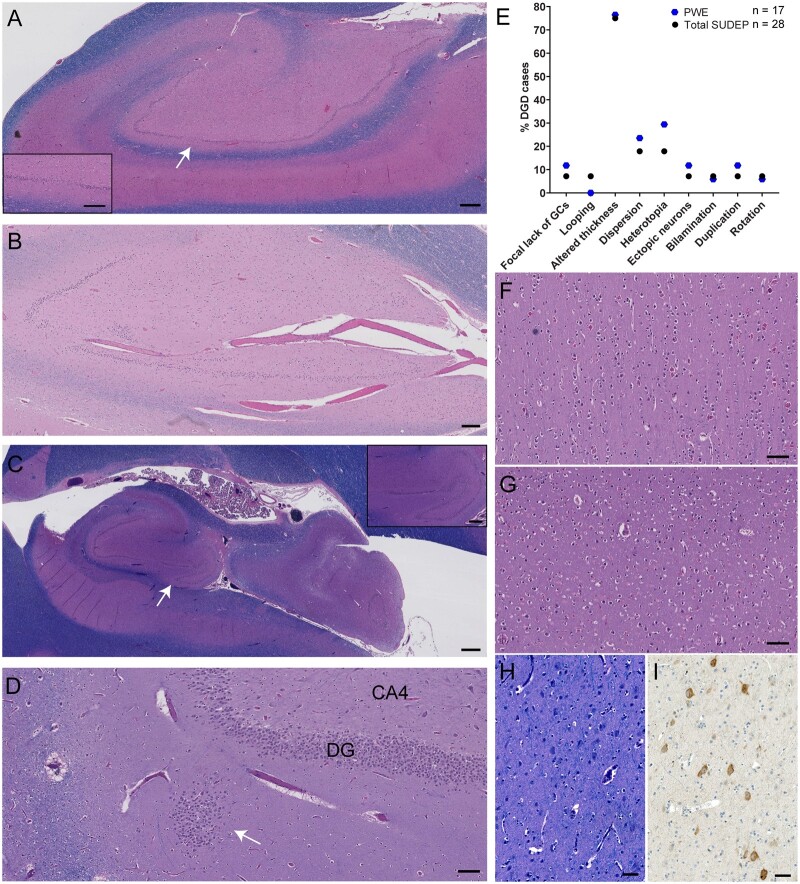
**DGD and FCD in epilepsy.** (**A**) Hippocampal section at the level of LGN shows an example of mild DGD, with variation in the thickness of the DG and focal loss of granule cells (white arrow indicates magnified inset) in a 32-year-old male, probable SUDEP case. Scale bar represents 500 µm, inset 300 µm. (**B**) Hippocampal section at LGN shows mild DGD with focal bilamination, focal loss of granule cells that is adjacent to blood vessels, and focal thinning of the DG in a 19-year-old male, definite SUDEP case. Bilamination was identified in 3/92 cases (2 definite SUDEP, 1 PWE). Scale bar represent 200 µm. (**C**) Hippocampal section at LGN shows DGD, with hippocampal rotation and variation in the thickness of the DG (white arrow indicates magnified inset) in a 24-year-old male, definite SUDEP case. Hippocampal rotation was identified in 3/92 cases (2 definite SUDEP, 1 PWE). Duplication of the DG was identified in 4/92 cases (2 definite SUDEP plus, 2 PWE). Scale bar represents 1 mm, inset 400 µm. (**D**) Hippocampal section at LGN shows DGD, with a focal heterotopia (white arrow) of granule cells in the molecular layer adjacent to the DG in a 21-year-old male, definite SUDEP case. This is the same case as in Fig. 3B. Scale bar represents 100 µm. (**E**) The percent of cases with DGD (*n* = 45 cases, excluding two possible SUDEP) are depicted for the specific DG findings described on the *x*-axis. The most frequent finding in both groups was altered thickness of the DG. (**F**) FCD IA with columnar arrangement of neurons in the insular cortex and disorganization of neurons in the temporal cortex (**G**) of a 34-year-old female, probable SUDEP case. Petechial haemorrhages are also seen in these cortical sections. Scale bars represent 100 µm. (**H and I**) FCD IIA with dysmorphic neurons and abnormal organization of neurons in the frontal cortex of a 20-year-old male, definite SUDEP case. Dysmorphic neurons are evident with immunostaining of phospho-S6 (Ser240/244). Scale bar represents 50 µm. Overall, there were 8/92 cases with FCD IA and 25/92 cases with FCD IIA. All sections in **A**–**D**, **F**–**H** were stained with LFB/H&E.

Histologic images of DGD and FCD are shown in Fig. 2. DGD is represented by mild DGD, mild disruption of the granule cells (GCs) in the dentate gyrus (DG) by blood vessels with GC loss and focal thinning of the DG, hippocampal rotation with variable DG thickness, and GC heterotopia (Fig. 2A–D). Hippocampal rotation was observed in 3.2% (1/31) of PWE and 3.6% (2/55) of SUDEP cases. DGD was the only neuropathological finding in 23.5% of PWE and 25.0% of SUDEP cases. Most cases with DGD in PWE and SUDEP groups had additional findings. The average number of DGD findings per case was 1.8 for PWE and 1.5 for SUDEP. The most frequent DG finding in PWE (76.5%) and SUDEP (75.0%) groups was altered DG thickness (*P* > 0.9, Fig. 2E). The second most frequent finding was heterotopia with 29.4% in PWE and 17.9% in total SUDEP (*P* = 0.5). There were no significant differences between the specific DG findings in PWE and in SUDEP cases with DGD. FCD occurred in 35.9% of the NASR cohort: 8 cases had FCD IA and 25 cases had FCD IIA. Average age of epilepsy onset in cases with FCD IA was 9.5 years of age (median 3 years of age, *n* = 7) from known information. In cases with FCD IIA, average age of epilepsy onset from known information (*n* = 16) was 16 years of age (median 10 years of age). A representative case with FCD IA in a probable SUDEP case with a clinical history of Lennox Gastaut syndrome, global developmental delay and autism showed an abnormal columnar organization of neurons in the insula cortex and disorganization in a section from the superior and middle temporal gyri (Fig. 2F and G). FCD IIA was observed on LFB/H&E sections, as well as on phospho-S6 immunostained sections with a range in positive neurons for each case. A representative case with FCD IIA is shown in a definite SUDEP case, with dysmorphic neurons and abnormal organization of neurons in the superior frontal gyrus (Fig. 2H and I).

**Figure 3:**
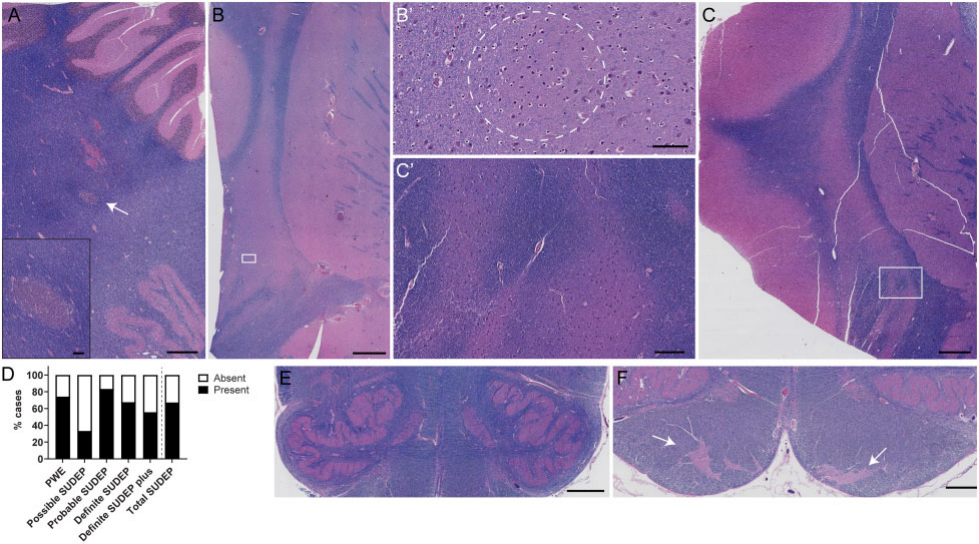
**Neurodevelopmental findings in epilepsy**. (**A**) Focal cerebellar heterotopia (white arrow) in the white matter adjacent to the dentate nucleus of a 31-year-old male, definite SUDEP case. Heterotopia is magnified further in the inset. A region of capillary telangiectasia can also be seen above the heterotopia. Scale bar represents 500 µm, inset 200 µm. (**B**) Heterotopia in the claustrum, in the form of an enlargement of the gray matter, with the appearance of ‘additional’ white matter, and dysmorphic neurons in a 21-year-old male, definite SUDEP case (white box indicates magnified inset in **B’** depicting abnormal cells within the dotted white circle). This is the same case as in Fig. 2D. Scale bar represents 500 µm, inset 100 µm. (**C**) Heterotopia in the claustrum, in the form of abnormal organization and enlargement of the gray matter in a 24-year-old female, definite SUDEP plus case (white box indicates magnified inset in **C’** depicting abnormal organization of gray and white matter). This is the same case as in Fig. 2H. Scale bar represents 500 µm, inset 250 µm. (**D**) The frequency of neurodevelopmental findings in each group indicates that there is no significant difference between the total SUDEP and PWE groups (Fisher’s exact test). Neurodevelopmental findings included DGD, FCD, heterotopias, medullary arcuate nucleus dysgenesis, olivary dysplasia/hypoplasia, cerebellar dysplasia and polymicrogyria. (**E**) Bilateral dysplasia of the medullary inferior olives, with focal abnormal folding of the inferior olive in a 33-year-old female, PWE case. In addition to this finding, other findings in this case included bilateral dysgenesis of the hippocampal dentate gyrus, multifocal heterotopia, cerebellar dysplasia and moderate hippocampal sclerosis. Overall, olivary dysplasia/hypoplasia was identified in 7/92 cases. Scale bar represents 500 µm. (**F**) Bilateral dysgenesis of the medullary arcuate nucleus, with abnormal localization and formation (white arrows) in a 47-year-old female, definite SUDEP case. Overall, findings involving the arcuate nucleus of the medulla were identified in 3/92 cases. Scale bar represents 1 mm.

With a positive correlation of age at death and disease onset among SUDEP cases (Supplementary Fig. S1B), we also compared neurodevelopmental findings (i.e. FCD, DGD, heterotopias, medullary arcuate nucleus dysgenesis, medullary olivary dysplasia/hypoplasia, cerebellar dysplasia and polymicrogyria) in PWE and SUDEP groups. These findings are summarized in Fig. 3. There were no differences in the frequency of neurodevelopmental findings in PWE and SUDEP groups. The number of neurodevelopmental findings per case relative to age at disease onset in the 0–16 year-old group (*n* = 46) was 1.7-fold higher than in the cohort with epilepsy onset after age 17 years (*n* = 25; *P* = 0.07). In addition to the hippocampal heterotopia (Fig. 2D), extra-hippocampal heterotopias are shown in Fig. 3A–C. Heterotopias were observed in 12.0% (11/92) of cases. A heterotopia in cerebellar white matter is depicted from a definite SUDEP case (Fig. 3A). Broadening of the claustrum with dysmorphic neurons or abnormally organized neurons was observed in two SUDEP cases (definite SUDEP and definite SUDEP plus), consistent with a heterotopia (Fig. 3B and C) and has not been frequently described in previous epilepsy studies although has been reported in an epilepsy patient and animal model.^47^^,^^48^

Because the brainstem contains autonomic nuclei implicated in SUDEP pathogenesis^1^^,^^10–14^ and detailed neuropathologic investigation of this region in SUDEP cohorts is limited,^4^^,^^14^^,^^33^ we reviewed the brainstem on microscopic examination by sampling midbrain (*n* = 91), pons (*n* = 83) and medulla (*n* = 83) in all cases. The medulla was sampled at 1–4 levels in each case when available, with 33.7% (31/92) of cases at 4 different levels within a 1 cm region above the obex (0.25 cm apart). A total of 16.5% (15/91) of NASR cases had brainstem findings. These findings were all described to be of unknown significance. Of these 15 cases, all included medullary findings. One case had pathology at all brainstem levels (Duret haemorrhages). In the remaining 14 cases, pathology was seen in the inferior olive (*n* = 10), arcuate nucleus (*n* = 4) and medullary infarct (one case had findings in both the arcuate nucleus and the inferior olives). These findings are depicted in Fig. 3E and F, including olivary dysplasia and dysgenesis of the arcuate nucleus. Brainstem findings were non-significantly more common in PWE at 25.8% (9/31) than SUDEP at 11.1% (6/54; *P* = 0.07).

### Neuropathological findings by seizure type

**Figure 4:**
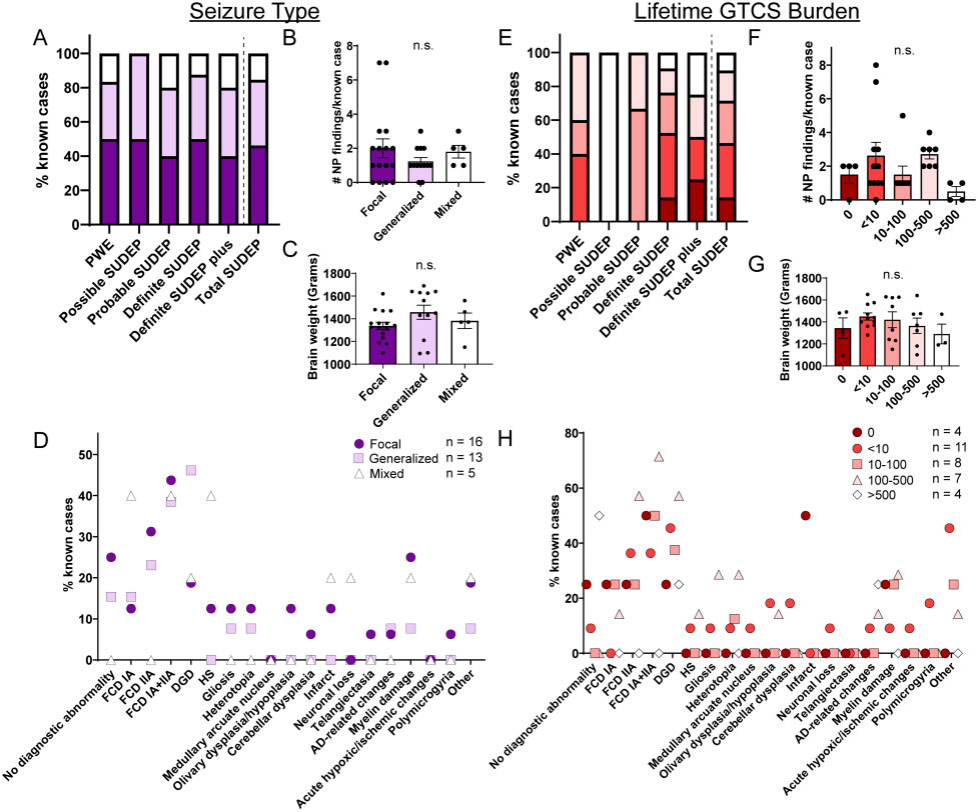
**Neuropathology analysed by seizure type and lifetime GTCS burden.** (**A**) The percent of known cases with focal only, generalized only, or mixed seizure types in epilepsy overall (regardless of cause of death; COD). (**B**) The number of neuropathology findings per case in each seizure type group is not significantly different (one-way ANOVA). (**C**) Brain weight in each seizure type group is not significantly different (one-way ANOVA). (**D**) The percent of cases with neuropathology findings in each seizure type group are depicted for the diagnoses described on the *x*-axis. For the focal only group, the most frequent finding was FCD (43.8%). For the generalized only group, the most frequent finding was DGD (46.2%). For the mixed seizure types group, the most frequent findings were FCD (40.0%) and hippocampal sclerosis (40.0%). (**E**) The percent of known cases with lifetime GTCS burden in epilepsy overall (regardless of COD). (**F**) The number of neuropathology findings per case in each GTCS group is not significantly different (one-way ANOVA). (**G**) Brain weight in each GTCS group is not significantly different (one-way ANOVA). (**H**) The percent of cases with neuropathology findings in each GTCS group are depicted for the diagnoses described on the *x*-axis. For the majority of the GTCS groups (10–100, 100–500), the most frequent finding was FCD. For the cases with <10 lifetime GTCS, the most frequent finding was DGD. For cases with >500 lifetime GTCS, the most frequent diagnosis was no abnormality. For the cases with 0 lifetime GTCS, the most frequent findings were FCD and infarct. The mean is depicted with errors bars representing SEM.

We compared neuropathological findings in groups with different seizure types (focal only, generalized only and mixed seizure types). The percentage of cases for each seizure type with available information (*n* = 34; 6 PWE, 2 possible SUDEP, 5 probable SUDEP, 16 definite SUDEP, 5 definite SUDEP plus) is shown in Fig. 4A. Total SUDEP and PWE groups had no significant difference in the frequency of a specific seizure type. The number of neuropathological findings per case and brain weights for each seizure type group was similar regardless of SUDEP status (Fig. 4B and C). The frequency of neuropathological findings in each group is depicted and summarized in Fig. 4D and Supplementary Table S2. Among cases with focal seizures only, FCD was the most frequent finding, present in 43.8% of cases with the FCD IIA type predominant (31.3%; Fig. 4D). In cases with generalized seizures only, the most frequent finding was DGD (46.2%), followed by FCD (38.5%). In cases with mixed seizure types, the most frequent findings were FCD and hippocampal sclerosis (HS) (each 40.0%). There was no significant difference in the percentage of FCD in focal seizure-only cases or DGD in generalized seizure-only cases compared to all cases, regardless of SUDEP status. The average frequencies of neurodevelopmental findings were 1.0 in focal cases, 0.9 in generalized cases, and 0.6 in mixed focal-generalized seizure cases (*P* > 0.05). Seizure type (2 focal, 1 mixed) information was available for only three cases with brainstem findings. The two cases with focal seizures had medullary inferior olive dysplasia and the case with mixed seizure types had a medullary ischaemic infarct. Due to the diversity of findings and small number of cases in each SUDEP subgroup, additional statistical analyses were not performed.

### Neuropathological findings by lifetime GTCS burden

Lifetime GTCS burden, associated with an increased SUDEP risk in some cases,^1^^,^^5^ was evaluated to determine whether there was an association with neuropathology findings. The percent of cases with GTCS among those with information is shown for each group (*n* = 34; 5 PWE, 1 possible SUDEP, 3 probable SUDEP, 21 definite SUDEP, 4 definite SUDEP plus) in Fig. 4E. There was no significant difference in PWE or SUDEP cases with >10 (60.0% PWE, 53.6% SUDEP) or >100 (40.0% PWE, 28.6% SUDEP) lifetime GTCS. Regardless of SUDEP status, there was no difference in the number of neuropathological findings per case or brain weight among the GTCS groups (Fig. 4F and G). The frequency of neuropathological findings in each group is depicted and summarized in Fig. 4H, Supplementary Table S3. Regarding neurodevelopmental findings, the average number of findings for each group was: 0 GTCS: 0.8, <10 GTCS: 1.6, 10–100 GTCS: 1.0, 100–500 GTCS: 1.7 and >500 GTCS: 0.3. There were too few cases with brainstem findings and seizure type data to make comparisons.

## Discussion

We identified neuropathological findings in 83.7% of the 92 epilepsy cases who died from SUDEP or other causes in NASR. Age at death was significantly younger for SUDEP cases than PWE and this correlated with an earlier age of epilepsy onset. We found similar frequencies of neuropathological findings in SUDEP cases and PWE, including DG, neurodevelopmental or brainstem findings.

Regarding potential SUDEP risk factors, we found similar frequencies of GTCS, polytherapy, sex and ASM adherence in PWE and SUDEP cases, as previously reported.^5^^,^^7–9^ The significantly younger age at death in SUDEP cases and positive correlation of age at death and epilepsy onset was reported.^5^ We found a significant negative correlation of brain weight and epilepsy duration in SUDEP cases, that was present but not significant in PWE cases. Reduced brain weight in SUDEP cases is not likely related to aging, because their age at death was younger than PWE, but more likely reflects greater brain atrophy in chronic epilepsy, potentially due to higher seizure burden, effects of ASMs, restricted neurodevelopmental growth or accelerated volume loss in SUDEP cases.^14^^,^^23–30^

We found no significant differences in the frequency of neuropathological findings in SUDEP cases compared to PWE, by neuropathologists blinded to the adjudicated COD, similar to an earlier neuropathology study of 145 SUDEP cases.^4^^,^^33^ This prior study^4^ excluded non-SUDEP epilepsy and had variable brain sampling (average 12.5 blocks/case, 76.6% of cases with whole brain fixation), included cases with an older age at death, and used inclusion criteria of possible/near/combined SUDEP for 34/145 cases. Both ours and this series included more male cases and commonly observed pulmonary pathology (e.g. oedema or congestion) (Table 4). Our NASR cohort had a higher percentage of cases with cortical malformations and normal neuropathology (two-fold), but lower percentages of brain swelling, HS (half), acute hypoxic-ischaemic change, and CNS tumours. Compared to our cohort with frequent DGD and FCD, Thom et al. (2016) described fewer cases with cortical malformations (Table 4): DG GC dispersion without HS (4%) and FCD (7.6%; including mild, I, IIB and IIID). A higher frequency of cortical malformations in our cohort may be consistent with the majority of cases having an epilepsy onset before 17 years of age, who had a 1.7-fold trending increase in neurodevelopmental findings. DG findings were described in epilepsy and in some cases of sudden unexplained death in childhood (SUDC) regardless of febrile seizure history, although the significance and specificity of hippocampal findings in general is unclear as they may result from seizures, contribute to seizure pathogenesis, or be an unrelated phenomenon.^42^^,^^49–54^ Hippocampal rotation was observed in 9.7% of the SUDEP cases in Thom et al. and in 3.6% of the SUDEP cases in our cohort. Hippocampal rotation occurs in 23% of the general population, predominantly on the left, as seen on MRI and is likely an incidental non-pathologic finding.^55^^,^^56^ Brainstem pathology was not described by Thom et al., but they noted potential compression in cases with increased brain swelling. Neuropathological findings in SUDC cases include potential neuronal migration defects of the rhombic lip, pertaining to findings in the cerebellum and brainstem.^51^ Brainstem findings in SUDC cases included medullary arcuate and olivary dysplasias, similar to our findings. However, these findings are not clearly related to cause of death or seizures. Hippocampal sclerosis was identified at a lower frequency in our cohort, potentially related to the differences seen in the clinical variables of the cohorts (on average disease duration was longer and with a wider range in Thom et al.). The cohort described by Thom et al. was compared to additional SUDEP cohorts, PWE post-mortem and surgical cases.^33^ These findings indicated no lesion type was over-represented in SUDEP. Furthermore, differences in the frequency of neuropathological findings across these series were reported and may potentially be due to the heterogeneity of cases and other clinical variables (e.g. SUDEP subgroups, epilepsy syndromes, epilepsy onset, epilepsy duration). Cardiopulmonary pathology was also reviewed with a range of severity in findings (Table 4), similar to previous studies.^33^^,^^57^

**Table 4:** Comparison of neuropathology findings to previous cohort

Series	Thom 2016^4^		NASR cohort	
Number of cases	145^a^		61^b^	
	Average	Range	Average	Range
Age at death range (years)	40.0	2–82	30.0	3–57
Age of onset	17.9	0–82	15.3	0–56
Disease duration	23.2	1–74	17.8	1–55
Male (% cases)	61.1%		65.6%	
Cases with whole brain fixed	76.6		100.0	
Cases with brain histology	100.0		100.0	
Brain swelling	28.3		0.0	
Microscopic findings	89.0		75.4	
Traumatic lesions	16.6		4.9	
Cortical malformations	14.5		42.6	
Vascular malformations			4.9	
Hippocampal sclerosis	21.4		11.5	
Old infarcts	6.9		9.8	
CNS tumors	13.0		0.0	
Cerebellar atrophy	40.7		0.0	
Hippocampal rotation	9.7		3.3	
Acute hypoxic-ischaemic change	55.2		1.6	
No pathology findings	11.0		24.6	
External findings/general PM examination				
Pulmonary findings	68.0		90.4	
Cardiac findings			63.5	

Cortical malformations include FCD, heterotopias and polymicrogyria. Vascular malformations include telangiectasia and venous angioma.

a One hundred and eleven without possible/near/combined SUDEP cases.

b Fifty-five without possible SUDEP.

In the NASR cohort, there was no detectable difference in glial populations in SUDEP and PWE as seen on LFB/H&E, regarding gliosis, myelin damage, etc. The molecular mechanisms underlying SUDEP risk have been investigated in brain tissue using immunohistology, genetic analyses, proteomics and RNA sequencing (RNAseq). In the medulla, differences were found in glial populations of SUDEP cases compared to epilepsy and non-epilepsy control groups: reduced cell density of vimentin-positive glia in the ventrolateral medullary region and subpial layer as well as connexin 43 (Cx43)-positive glia in the medial raphe, implicating glial cells in impaired respiratory regulation.^40^ MRI studies found reduced brainstem volume in SUDEP cases.^14^ Another study correlated MRI findings with immunohistochemistry markers microtubule associated protein 2 (MAP2), myelin basic protein (MBP) and synaptophysin (SYP) in several medullary regions,^37^ finding differences between epilepsy and non-epilepsy controls but no differences between SUDEP and epilepsy cases. Markers of inflammation, gliosis, acute neuronal injury due to hypoxia and blood brain barrier disruption were evaluated in the hippocampus, amygdala and medulla of 58 cases.^34^ They found reduced numbers of CD163+ macrophages in the medulla in SUDEP cases and lower IgG present in hippocampal subsector CA1 and in parahippocampal gyrus compared to controls, but no clear immunohistochemical signature as identified with the markers investigated. Two additional studies found no differences between PWE and SUDEP with several markers in medullary regions.^35^^,^^36^ Whole exome sequencing in surgical brain tissue from epilepsy patients that died from SUDEP implicate several gene variants that may increase risk of SUDEP.^58^^,^^59^ We recently identified a number of protein differences between epilepsy and non-epilepsy control cases by localized proteomics in cortex and hippocampal CA1-3 and DG,^60^ however found no significant protein differences when comparing SUDEP to epilepsy and few differences in the hippocampus of high-risk SUDEP MTLE cases with a prolonged PGES by RNAseq.^39^ Future studies should validate molecular markers to better understand SUDEP risk and to potentially identify novel markers in brain tissue.

Our study had several limitations. Potential pathogenic gene variants were not assessed in this cohort. Our analyses were based on NASR referrals, skewed by major referral sources: the San Diego Medical Examiner Office (mainly low socioeconomic white and Hispanic patients) and direct referrals (mainly high socioeconomic white patients). In addition to a limited number of cases for some SUDEP subgroups, some analyses were limited to cases with available information, reinforcing the importance of banking brain tissue from well characterized SUDEP cases. This is relevant because SUDEP cases in our NASR cohort did not necessarily have SUDEP risk factors and additional cases may allow for studies among specific epilepsy syndromes and for molecular studies when FFPE or frozen tissue is available. Finally, larger consensus studies among different pathologists should evaluate the frequency of neuropathological findings and their significance in the context of epilepsy when compared with non-epilepsy control cases, as our and other recent studies have observed some similar pathological findings in control cases and inconsistency of observations my multiple blinded reviewers.^49^^,^^53–56^

Overall, few neuropathological differences were identified between SUDEP and PWE in the NASR and other cohorts. Neuropathology may be useful as a tool in conjunction with clinical history and MRI for identifying epilepsy-related pathology, but in most cases neuropathological findings have not provided insight on SUDEP pathophysiology. Further investigation is warranted in larger numbers of non-SUDEP epilepsy and SUDEP cases with known case information to explore neuropathological findings in different epilepsy syndromes and to identify molecular markers of SUDEP risk or pathogenesis, particularly in the brainstem.

## Supplementary Material

fcab192_Supplementary_Data

## Abbreviations

AD-related changes =: Alzheimer’s disease-related changes
ASM =: anti-seizure medication
BMI =: body mass index
COD =: cause of death
DGD =: dentate gyrus dysgenesis
FCD =: focal cortical dysplasia
FFPE =: formalin fixed paraffin embedded
GC =: granule cell
GTCS =: generalized tonic-clonic seizure
HS =: hippocampal sclerosis
LFB/H&E =: luxol fast blue/haematoxylin and eosin
NASR =: North American SUDEP Registry
PGES =: postictal generalized EEG suppression
phospho-S6 =: Ser240/244 phosphorylated ribosomal protein S6
PMI =: post-mortem interval
PWE =: people with epilepsy
SIDS =: sudden infant death syndrome
SUDC =: sudden unexplained death in childhood
SUDEP =: Sudden unexpected death in epilepsy
