# Supplementary material for: Neuropathology in the North American sudden unexpected death in epilepsy registry
Source: Brain Commun. 2021 Aug 23;3(3):fcab192. doi: 10.1093/braincomms/fcab192 (PMC8417454; doi:10.1093/braincomms/fcab192)

Supplementary materials:

Supplemental Table 1. Anti-Seizure Medications

Supplemental Table 2. Neuropathology by Seizure Type

Supplemental Table 3. Neuropathology by Lifetime GTCS Burden

Supplemental Figure 1. Case history and correlation analyses. A) Age at death (years) was significantly lower in the total SUDEP group when compared to PWE ($p = 0.006$, two-tailed t-test). When evaluating by the SUDEP subgroups, the definite SUDEP group was significantly younger than the PWE group ($p = 0.01$) and the possible SUDEP group ($p = 0.02$, one-way ANOVA Tukey's post-hoc test). The mean is depicted with errors bars representing SEM. **B)** Overall in epilepsy there was a significant positive correlation of age at death (years) and age of epilepsy onset (years, $p < 0.0001$, $R^2 = 0.2$, Pearson correlation). When evaluating by COD, there was a significant positive correlation in the total SUDEP group ($p = 0.0005$, $R^2 = 0.2$), no correlation in the PWE group, and no significant difference between the two correlations. **C)** Overall in epilepsy there was a significant positive correlation of age at death (years) and epilepsy duration (years, $p = 0.0003$, $R^2 = 0.2$). When evaluating by COD, there was a significant positive correlation in the PWE group ($p = 0.04$, $R^2 = 0.2$), total SUDEP group ($p = 0.007$, $R^2 = 0.2$), and no significant difference between the two correlations. **D)** Overall in epilepsy, there was a significant negative correlation of brain weight (grams) and age at death ($p = 0.03$, $R^2 = 0.05$). When evaluating by COD, there was no correlation. **E)** Overall in epilepsy, there was no correlation of brain weight (grams) and age of epilepsy onset. When evaluating by COD, there was also no correlation. **F)** Overall in epilepsy, there was no correlation of brain weight (grams) and epilepsy duration. When evaluating by COD, there was a significant negative correlation in the total SUDEP group ($p = 0.05$, $R^2 = 0.09$), no correlation in the PWE group, and no significant difference between the two correlations. The 95% confidence intervals for the Pearson correlations are depicted by dashed lines.

Supplemental Table 1. Anti-Seizure Medications

Study Group	None	1 ASM	2 ASM	3+ ASM
PWE	8.7 %	26.1 %	30.4 %	34.8 %
Possible SUDEP	25.0	75.0	0.0	0.0
Probable SUDEP	0.0	50.0	33.3	16.7
Definite SUDEP	16.2	32.4	35.1	16.2
Definite SUDEP plus	25.0	25.0	0.0	50.0
Total SUDEP*	15.7	33.3	29.4	21.6

*Excludes possible SUDEP. Includes cases with known information, n = 78.

Supplemental Table 2. Neuropathology by Seizure Type

Neuropathology Finding	Focal (n=16)	Generalized (n=13)	Mixed (n=5)
No diagnostic abnormality	25.0%	15.4%	0.0%
FCD IA + IIA	**43.8**	38.5	**40.0**
FCD IA	12.5	15.4	40.0
FCD IIA	31.3	23.1	0.0
DGD	18.8	**46.2**	20.0
HS	12.5	0.0	**40.0**
Gliosis	12.5	7.7	0.0
Heterotopia	12.5	7.7	0.0
Medullary arcuate nucleus dysgenesis	0.0	0.0	0.0
Medullary olivary dysplasia/hypoplasia	12.5	0.0	0.0
Cerebellar dysplasia	6.3	0.0	0.0
Infarct	12.5	0.0	20.0
Neuronal loss	0.0	0.0	20.0
Telangiectasia	6.3	0.0	0.0
AD-related changes	6.3	7.7	0.0
Myelin damage	25.0	7.7	20.0
Acute hypoxic/ischemic changes	0.0	0.0	0.0
Polymicrogyria	6.3	0.0	0.0
Other	18.8	7.7	20.0

Bolded are the most frequent percent of findings within each group. Includes cases with known information, n = 34.

Supplemental Table 3. Neuropathology by Lifetime GTCS Burden

Neuropathology Finding	0 (n=4)	<10 (n=11)	10-100 (n=8)	100-500 (n=7)	>500 (n=4)
No diagnostic abnormality	25.0%	9.1%	0.0%	0.0%	**50.0%**
FCD IA + IIA	**50.0**	36.4	**50.0**	**71.4**	0.0
FCD IA	25.0	0.0	25.0	14.3	0.0
FCD IIA	25.0	36.4	25.0	57.1	0.0
DGD	25.0	**45.5**	37.5	57.1	25.0
HS	0.0	9.1	0.0	14.3	0.0
Gliosis	0.0	9.1	0.0	28.6	0.0
Heterotopia	0.0	9.1	12.5	28.6	0.0
Medullary arcuate nucleus dysgenesis	0.0	9.1	0.0	0.0	0.0
Medullary olivary dysplasia/hypoplasia	0.0	18.2	0.0	14.3	0.0
Cerebellar dysplasia	0.0	18.2	0.0	0.0	0.0
Infarct	**50.0**	0.0	0.0	0.0	0.0
Neuronal loss	0.0	9.1	0.0	0.0	0.0
Telangiectasia	0.0	0.0	0.0	0.0	0.0
AD-related changes	0.0	9.1	0.0	14.3	25.0
Myelin damage	25.0	9.1	25.0	28.6	0.0
Acute hypoxic/ischemic changes	0.0	9.1	0.0	0.0	0.0
Polymicrogyria	0.0	18.2	0.0	0.0	0.0
Other	0.0	**45.5**	25.0	14.3	0.0

Bolded are the most frequent percent of findings within each group. Includes cases with known information, n = 34.

Supplementary Figure 1.

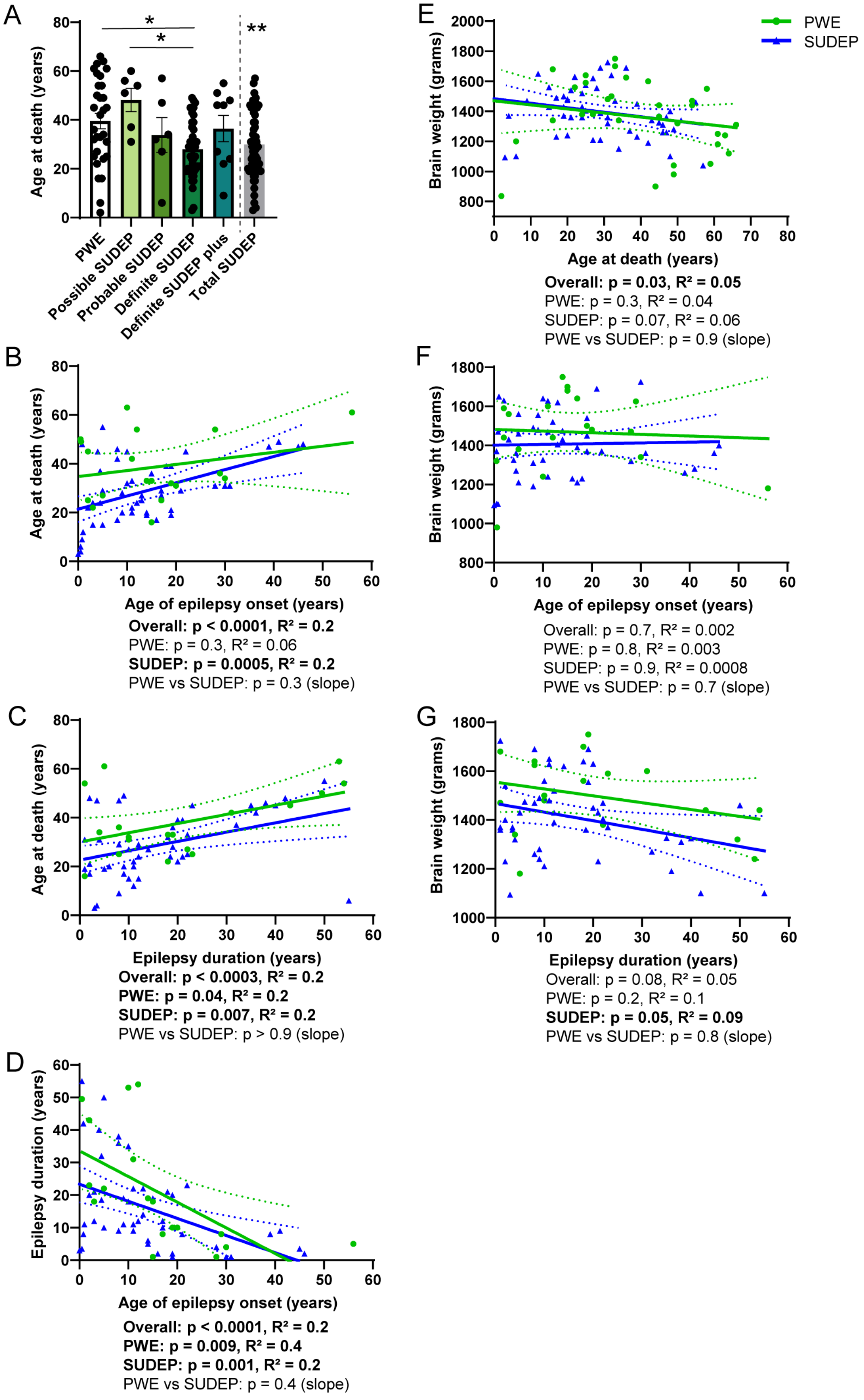

Supplement: fcab192_Supplementary_Data [file fcab192_supplementary_data.pdf]
